# Patent pledges, open IP, or patent pools? Developing taxonomies in the thicket of terminologies

**DOI:** 10.1371/journal.pone.0221411

**Published:** 2019-08-20

**Authors:** Jonas Fabian Ehrnsperger, Frank Tietze

**Affiliations:** Centre for Technology Management, Innovation and Intellectual Property Management (IIPM), Institute for Manufacturing, University of Cambridge, Cambridge, United Kingdom; Wuhan University, CHINA

## Abstract

Recently, a range of organisations, including car and consumer electronics manufacturers, have applied so-called patent pledges. A patent pledge is a publicly announced intervention by patent-owning entities (‘pledgers’) to out-license active patents to the restricted or unrestricted public free from or bound to certain conditions for a reasonable or no monetary compensation. Despite growing research to better understand this phenomenon, the underlying terminology remains contradictory. We apply an inductive research approach using qualitative coding to analyse 60 patent pledges made by 80 organisations. Based on this analysis, we propose a three-dimensional taxonomy that distinguishes eight types of patent pledges. Extending this taxonomy using case examples, we then propose a generalised patent licensing taxonomy. This second taxonomy can be used to distinguish patent licensing strategies, including other frequently used approaches, such as patent pools and cross-licenses. Finally, we use the patent pledge taxonomy to illustrate how patent owners change their licensing strategies over time and how it can support strategic decision processes within an organisation. We contribute to the field of patent management by building an ontology of patent pledges through proposing a definition and eight types. The patent licensing taxonomy enables organisations to devise and choose licensing strategies, and to illustrate licensing approaches of competitors, for instance.

## Introduction

Existing theories explaining why and how organisations use Intellectual Property Rights (IPRs), such as those considering *revenue* and *profit dissipation effects* hardly explain why innovative firms such as Tesla Motors give away patents for free (i.e. without demanding royalty payments) [1]. While these effects and further theories play an important role in the licensing literature, the increasingly complex business environment and certain novel licensing approaches require the existing research to be adapted and extended.

The rise of open innovation and open source software caused some organisations to rethink long-established practices, including their attitude towards IP strategies [2]–[4]. Subsequently, the announcement made by Tesla in 2014 triggered an ongoing debate inside and outside the academic world about what has been often labelled an *open IP strategy* more broadly or *patent pledge* specifically. While Tesla’s patent pledge seems the mostly widely discussed pledge to date, it is by far not the only one. Other lesser-known examples for freely made available IPRs include the QR code technology. Even though such pledges have grown in frequency, they are far from new [5], [6]. Historic examples from the early 2000s include IBM’s pledge not to assert 500 of its patents against the development, distribution and use of open-source software. Hence, the rise of patent pledges suggests a demand for more research.

This demand has been recognised by researchers, which resulted in some seminal works in the area of patent pledges [7]. The increasing research, however, has created ambiguity: academics and industry professionals are inconsistent in their use of terms such as patent pledges and open IP. For many, only IPRs that are free of charge and available to the unrestricted public constitute a patent pledge. Others adopt a broader view by considering those to be pledges where access to IPRs are available not for free, but for reduced licensing-fees. This inconsistency hinders the comparison of academic work and impedes communication between IP professionals. Furthermore, whilst many scholars use the notion of *IP pledges*, they generalise across all IPRs. However, since distinct IPRs protect different kinds of intellectual assets by law, it is too great a leap to assume that they all entail the same licensing strategies. For instance, it appears reasonable to assume that organisations apply different licensing strategies for patents (that protect technical inventions for a maximum of 20 years) and trademarks (that protect recognisable expressions that can theoretically be protected forever).

This paper addresses these problems by first proposing an inductively derived definition for patent pledges; second, by proposing a taxonomy of patent pledges consisting of eight mutually exclusive types; third, by proposing a patent licensing taxonomy that puts patent pledges into perspective to other common licensing types, such as cross-licenses and patent pools. The taxonomy of patent pledges emerges directly from patent licensing statements, whereas the patent licensing taxonomy constitutes an extension of the patent pledge taxonomy, and is validated using case studies. With these taxonomies, we aim to facilitate the distinction between different types of patent pledges and other patent licensing strategies by positioning them in relation to each other, making it possible to recognise differences as well as similarities. Hence, this paper contributes to the ontology for the patent licensing theory. As we focus only on patents, we provide a detailed overview of patent licensing that does not generalise across all types of IPRs.

This paper is structured as follows: section 2 summarises existing research on patent pledges contrasting existing definitions. Section 3 describes the data collection and qualitative coding process, which lay the groundwork for the taxonomy development. In section 4 we elaborate a definition of patent pledges and describe the patent pledge taxonomy and the patent licensing taxonomy. Section 5 discusses the findings, specifically focusing on changing licensing approaches of case examples within the context of the patent licensing taxonomy, and also emphasises limitations and suggestions for future research. Lastly, section 6 concludes by summarising the contributions of this paper.

## Theoretical background

Patent pledges can be interpreted as a phenomenon that has only gained interest during the past decade. Despite its importance in both the legal and economic literature, it has not been formally studied until 2012 [6]. However, patent pledges do not appear to be a new phenomenon. Burnett provides examples of organisations that applied what he calls ‘*forfeiture actions’* dating back to the 1940s [5]. These actions exhibit similarities to what is currently labelled a *patent pledge*, including the reasonable price for the respective IPRs and its broad availability. Despite this early occurrence of patent pledges in the business environment, its focus in the literature remained scarce. In 1983, Allen became one of the first scholars to specifically investigate the free exchange of knowledge between firms [8]. Whilst not explicitly referring to the notion of patent pledges, he describes the *‘free exchange of information about new techniques and plant designs among firms’* as a requirement for collective invention ([8], pg. 2). 20 years later, Harhoff, Henkel, and Hippel followed up on this idea by defining ‘*the free revealing of information by a possessor as the granting of access to all interested agents without imposition of any direct payment’* ([9], pg. 1753).

After these early works, an increasing but small number of scholars turned to patent pledges. According to the definition respective scholars use, the modern research can be broadly divided into two ‘camps’. On the one hand, some define patent pledges strictly as the access to patents without any monetary compensation. For example, Ziegler, Gassmann, and Friesike state that ‘*Patent release or give away for free means that in contrast to classic licensing and cross-licensing agreements*, *there is no contractual definition of compensation from the receiving end to the original patent holder*’ ([10], pg. 19). Similar definitions are provided by Raasch, Herstatt, and Balka, Schultz and Urban, Alexy, George, and Salter, Asay, Sundaresan, Jena, and Nerkar and Contreras, Hall, and Helmers [11]–[16]. Meanwhile, a second group widen the definition of patent pledges, including the access to patents on reasonable royalty-rates. For instance, Chander and Sunder broaden their definition of a *public domain* by adding the option to demand a nominal fee rather than no monetary compensation at all [17]. This idea is supported by Contreras and Contreras and Jacob [7], [18]. Chesbrough promotes this definition specifically in the context of Open Innovation [19].

Table 1 contrasts previous patent pledge definitions. It is important to note, however, that not all scholars specifically use the term patent pledges. In order to retain validity, we evaluated the content of definitions rather than the terminology itself.

**Table 1:** Existing patent pledge definitions.

Definitions including monetary compensation	Definitions excluding monetary compensation
*‘Resources for which legal rights to access and use for free (or for nominal sums) are held broadly*.*’* [17], pg. 1338	‘*Patent release or give away for free means that in contrast to classic licensing and cross-licensing agreements*, *there is no contractual definition of compensation from the receiving end to the original patent holder*.’ [10], pg. 19
*‘These pledges encompass a wide range of technologies and firms*: *from promises by multinational corporations like IBM and Google not to assert patents against open-source software users; to commitments by developers of industry standards to grant licenses on terms that are fair*, *reasonable*, *and non-discriminatory (FRAND)* …*’* [18], pg. 787	*‘OSI [Open Source Innovation] is characterised by free revealing of information on a new design with the intention of collaborative development of a single design or a limited number of related designs for market or non-market exploitation*.*’* [11], pg. 2
*‘Pledge commitments fall into three general categories*: *(1) the primary commitment to license patents*, *either on royalty-free or FRAND terms*, *or not to assert patents at all* …*’* [7], pg. 13	*‘Patent pledges are promises by patent holders not to enforce their patents under certain conditions*.*’* [12], pg. 30
	*‘* …*selective revealing as the voluntary*, *purposeful*, *and irrevocable disclosure of specifically selected resources*, *usually knowledge based*, *which the firm could have otherwise kept proprietary*, *so that they become available to a large share or even all of the general public*, *including competitors*.*’* [13], pg. 272
	*‘Parties are increasingly engaging in “patent pledging*,*” a phenomenon where parties voluntarily commit to limit enforcement of their patent rights*.*’* [14], pg. 261
	*‘An open IP strategy is any strategy that allows external inventors and firms the use of technology developed by the focal firm without any financial or cross-licensing obligation*.*’* [15], pg. n.a.
	‘ …*under a pledge model*, *patent assets are retained by their owners*, *who continue to incur maintenance and other fees*, *but the offensive use of such patents is significantly curtailed*.*’* [16], pg. 1

The contrasting definitions indicate that different types of patent pledges may exist. Contreras and Jacob distinguish between the price and its underlying calculation. They describe three broad categories [7]: *Primary Access Commitments*, *Secondary Royalty Commitments* and *Non-royalty Commitments* of patent owners. While this empirically derived categorisation is, to our knowledge, the first attempt towards a taxonomy of patent pledges, it also includes general promises that do not aim to facilitate access to IPRs, such as the pledge not to transfer patents to non-practicing entities (NPEs) [20]. Also, the authors’ categorisation involves the commitment to facilitate prior-art searches, and this is not directly related to patent licensing.

It is reasonable to conclude that academics previously supported two patent pledge definitions: (i) pledges that demand a reasonable fee for the usage, and (ii) pledges that do not demand any monetary compensation at all. This inconsistency impedes the comparison of different results, leading to a contorted understanding of patent pledges.

## Research approach

### Data collection

Similar to the analysis of Lerner, Strojwas and Tirole, we collected and coded official licensing agreements [21]. First, we had to decide on which patent initiatives to include in this study, i.e. which could be classified as patent pledges. As a starting point, we used the patent pledge dataset published by Contreras [22]. This dataset is, to our knowledge, the only publicly available dataset that includes patent pledges. The dataset lists 178 pledges and includes accompanying documents in which organisations pledge to apply a range of specific IP practices. However, the entries on this list are organisation-specific: every organisation that pledges a specific IP practice is counted as one entry (apart from the *Open Invention Network*). In order to avoid the inclusion of identical pledges, we consolidated the organisations that took part in the same initiative—counting pledges rather than individual organisations. To further filter the data, we excluded 13 entries: (1) the facilitation for prior-art searches (‘The Clearing House’, Microsoft, Yahoo, SAS), (2) the promise to enable/improve the community review of IPRs (IBM), (3) entries with vague formulations (Novell’s patent policy from 2014, Allergan’s social contract with patients), (4) the promise not to sell IPRs to non-practicing entities (Verizon, Cisco Systems), (5) entries with a lack of information (John Gilmore, ‘*Patent Licensing Principles*’ by Conversant), (6) an entry with the strict restriction to qualified customers (Microsoft’s Azure IP Advantage programme), (7) the mere statement of availability of licenses without the specification of standardized or reasonable terms (Microsoft). Furthermore, we added *The GreenXchange initiative*, *the OpenPOWER foundation* and patents relating to the QR-Code technology, because they specifically address the reasonable or free availability of patents based on standardised terms.

In total, our dataset comprised 60 patent pledges across 80 organisations (the *Open Invention Network*, the *OpenPOWER foundation*, the *Eco-Patent Commons*, *and* the *GreenXchange* are counted as one organisation; Ericsson, Sony, Sony Ericsson, Nokia, Siemens, Nokia Siemens Networks are counted separately.) The full dataset includes about 260 pages of DIN-A4 PDF-files. Only in the cases of GreenXchange and the Eco-Patent Commons (both no longer provide an active website) do we rely on empirical data and descriptions provided elsewhere [16], [23]. The complete list with information about number, type and area of technology is provided in S1 Table. It is important to note, however, that in some cases several patent pledges are announced in one initiative, which results in fewer dataset files than counted patent pledges. For instance, the patent pledge ‘Microsoft 03.12.2003’ is one PDF-file in which the firm announces three different patent pledges.

### Data analysis

**Fig 1:**
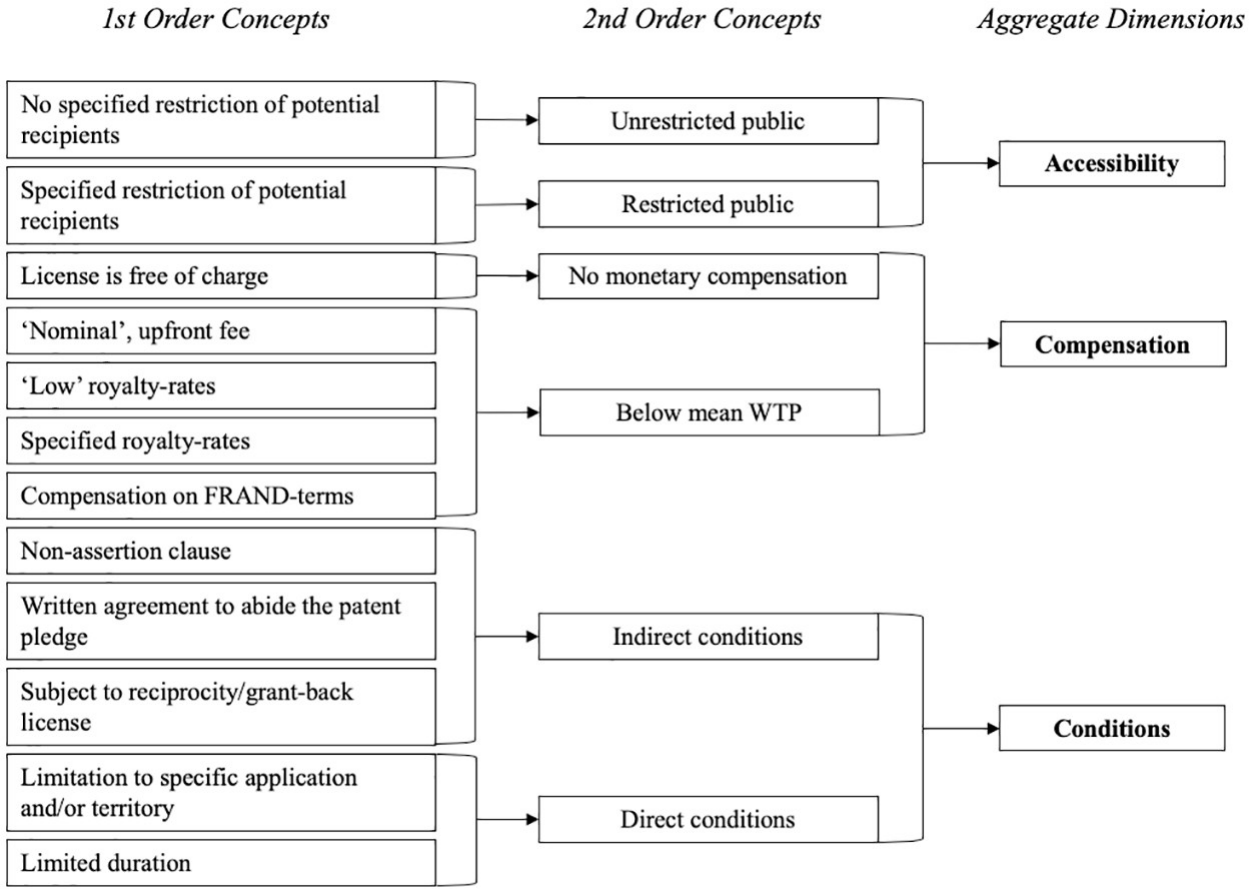
Coding process and emerging dimensions.

We coded the data in two cycles using NVivo software. While qualitative coding can be conducted manually, we used the software to facilitate the process of deleting, subsuming and recoding initial codes, and to facilitate the analysis. We did, however, find the raw data straightforward and consistent—we were only required to occasionally change codes. In a first coding cycle, we followed the qualitative analysis approach of conventional content analysis. We therefore avoided preconceived categories by allowing common themes to emerge from the raw data [24], [25]. Through initial coding we remained open to new interpretations by limiting the influence of our own subjective judgement [26], [27]. However, as suggested in the literature, we noted tentative ideas for themes that naturally arise through the human inclination of pattern-seeking [27]. In a second coding cycle, specifically through pattern coding, we revisited the data to reorganise and adapt initial concepts [27]. We structured the emerging concepts by summarising them into 1st order codes, 2nd order codes and aggregate dimensions, as suggested by [28], [29]. Three dimensions emerged from the coding: *Accessibility*, *Compensation* and *Conditions* (see Fig 1).

### Taxonomy development

The terms *taxonomy* and *typology* are often used interchangeably in the literature [30]. A difference between these terms is that the former consists of mutually exclusive sets while the latter relates to a set of ideal types. Crucially, only taxonomies provide key decisions that allow the classification of different organisations [31]. We avoid the common problem of summarising existing research in typology-based theory-building by using data that has not been analysed [32]. Since the proposed dimensions emerged directly from the dataset, we are confident that they are representative. Furthermore, they fulfil the criteria of *parsimony*, which Nickerson, Varshney and Muntermann describe as the manageable number of dimensions to prevent overextension [30]. At the same time, however, the dimensions allow for a clear distinction from each other. All elements of our dataset express forms of *Accessibility*, *Compensation* and *Conditions*, and this makes the taxonomy comprehensive [30].

## Results

### Definition of patent pledges

We propose the following definition of patent pledges that consists of the three elements that we derived from the content analysis of our dataset: *Accessibility*, *Compensation* and *Conditions*. It should be noted that we only consider active patents and pledges that are publicly announced (i.e. the unrestricted public can access the information).

*‘A patent pledge is a publicly announced intervention by patent owning entities (‘pledgers’) to out-license active patents to the restricted or unrestricted public free from or bound to certain conditions for a reasonable or no monetary compensation using standardized written or social contracts*.*’*

The restriction to active patents contrasts with patent donations, which generally refer to abandoned rights. The term *reasonable* in this context refers to fair, reasonable and non-discriminatory terms (FRAND-terms). We follow the definition of FRAND-terms described by [33]. There exist different approaches to calculate FRAND-terms. For instance, they can be economically determined by describing a hypothetical scenario of negotiations between the licensor and the licensee. A reasonable royalty-rate falls somewhere between the patent holder’s minimum fee they are willing to accept and the would-be infringer’s maximum willingness to pay (WTP) [34]. We follow the definition of WTP, or reservation price, described in the literature [35]–[37]. A reasonable royalty-rate is considered lower than the maximum WTP by the potential licensee, which is an important characteristic of our second taxonomy: the patent licensing taxonomy. The maximum WTP can be averaged among the elements of interest, which results in the *mean WTP*. Many studies apply mathematical and statistical methods to calculate the mean WTP for a specific business area [38], [39]. This value serves as a directive for a rough estimate of common royalty-rates in a specific industry. For example, the average royalty-rate in the automotive industry is given as 4.7% of the sales price, whereas in pharmaceuticals it is estimated to be 7.0% [40]. While the proposed taxonomies attempt to be general rather than industry-specific, we leave aside the calculation of mean WTP values for distinct business areas.

### Patent pledge taxonomy

For the taxonomy development, we applied a dichotomous scale to each of the three aggregate dimensions. This results in a taxonomy with eight types. Subsequently, we describe each dimension and the values it can take.

**Accessibility** is a measure for the potential recipients of the respective patents. Our analysis shows that patent pledges either address a large number of third parties or the public in general. We therefore suggest dividing this dimension into the two categories of *Restricted Public* and *Unrestricted Public*. Significantly, the *Restricted Public* category is only concerned with the a priori restriction to specified licensees and must not be confused with the dimension *Conditions* described below. This can be illustrated by the following example. Toyota pledges the availability of specified patents only ‘*to automakers who will produce and sell fuel cell vehicles*, *as well as to fuel cell parts suppliers and energy companies*’. This patent pledge restricts the number of potential licensees from the outset. On the other hand, IBM’s patent pledge from January 2005 does not restrict licensees a priori, but the use of their IPRs is subject to a specified condition: ‘*IBM hereby commits not to assert any of the 500 U*.*S*. *patents listed below*, *as well as all counterparts of these patents issued in other countries*, *against the development*, *use or distribution of Open Source Software*.’ This distinction is essential, since in the former example only specified third parties can make use of the patent pledge (i.e. restricted public). Whilst in the latter, anyone—subject to specific conditions—can access the patents (i.e. unrestricted public).

**Compensation** refers to the direct monetary compensation the pledger demands in exchange for the license. Our analysis reveals that patent pledges are either made in return for a *reasonable compensation* or *free of charge*. As outlined above, a reasonable compensation in the literature is considered to be below the mean WTP. Therefore, we suggest distinguishing this dimension in the two categories *Below mean WTP* and *None*.

**Conditions** is a measure for any usage-related condition for the respective patents. Our analysis shows that 42 out of 60 patent pledges are subject to explicitly mentioned conditions. The coding process revealed five non-exclusive categories of conditions listed in Table 2 below. We further distinguish the conditions according to the influence they have on the respective patent-usage. We suggest to summarise conditions that restrict the usage in either space (physical or technological) or time as **direct conditions**. In our sample, direct conditions are the restriction to a specific technology field/territory or a time limitation for the license. In contrast, **indirect conditions** do not affect the patent-usage in either space or time, such as the promise not to assert patents against the licensor (non-assertion clause).

**Table 2:** Patent pledge conditions.

	Condition	Example
**Direct conditions**	**Limitation to specific applications and/or territory** (in 25 out of 60 pledges)	*‘The policy also broadens Microsoft’s commitment to provide the academic community with IP under royalty-free terms for noncommercial use*.*’* Source: Microsoft pledge, March 2003
	**Limited duration** (in 3 out of 60 pledges)	*‘Patents related to fuel cell vehicles will be available for royalty-free licenses until the end of 2020*.*’* Source: Toyota pledge, 2015
**Indirect conditions**	**Non-assertion clause** (in 21 out of 60 pledges)	*‘A party is “acting in good faith” for so long as such party and its related or affiliated companies have not*: *asserted*, *helped others assert or had a financial stake in any assertion of (i) any patent or other intellectual property right against Tesla or (ii) any patent right against a third party for its use of technologies relating to electric vehicles or related equipment …’* Source: Tesla Motors pledge from 2014, Status: February 2019
	**Subject to reciprocity/ grant-back license** (in 10 out of 60 pledges)	‘*Qualcomm has had a long standing policy of broadly offering to license its standards essential patents for CDMA-based telecommunications standards on terms and conditions that are fair*, *reasonable*, *and free from unfair discrimination (FRAND)*, *subject to reciprocity*.*’* Source: Qualcomm pledge, 2008
	**Written agreement to abide the patent pledge** (in 1 out of 60 pledges)	*‘Thus*, *Google will require any person or entity to whom it sells or transfers any of the Pledged Patents to agree*, *in writing*, *to abide by the Pledge and to place a similar requirement on any subsequent transferees to do the same*.*’* Source: Google open patent non-assertion pledge. Status: February 2019

**Fig 2:**
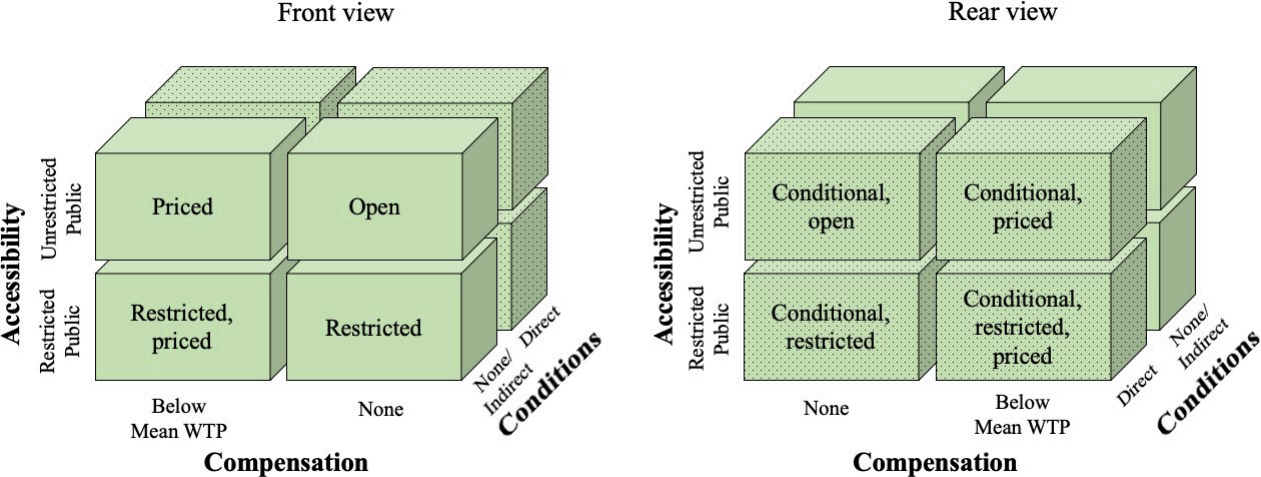
Patent pledge taxonomy.

Fig 2 shows the proposed patent pledge taxonomy. Subsequently, we describe each of the eight pledge types by providing an example. In this description, we do not add the *Conditions* dimension, since the conditions described in Table 2 can be equally applied to all types of patent pledges.

A **restricted, priced patent pledge** is a pledge that makes patents available to the restricted public in exchange for a reasonable price. An example for this type is iBiquity’s pledge from 2005, in which the organisation commits to license patents on FRAND-terms for *‘someone who is skilled in the art to manufacture NRSC-5 compliant transmission devices’*. The firm does not provide a description of these required skills, which leaves it open for interpretation.

A **priced patent pledge** is a pledge made from patent owning entities by announcing the general willingness to out-license on FRAND-terms. Significantly, the compensation *Below Mean WTP* must be equally available to all interested parties. An example is the patent pledge from NTT DoCoMo et al. from 2002, in which the companies pledge the availability of licenses relating to the W-CDMA technology to the unrestricted public for ‘*a cumulative royalty-rate below 5%’*.

A **restricted patent pledge** describes the free availability of licenses for the restricted public. The merger *thepatentpledge*.*org* is an example of this type in which 35 organisations pledge not to assert their software patents against firms that employ less than 25 people [41]. Another example is Toyota’s patent pledge from 2015, in which the firm announced that patents relating to fuel-cell technology would be freely available to ‘*automakers who will produce and sell fuel cell vehicles*, *as well as to fuel cell parts suppliers and energy companies who establish and operate fuelling stations*’.

An **open patent pledge** constitutes patents that are freely available to the unrestricted public. Tesla Motors’ pledge from 2014, for instance, is of this type. Even though there has been much criticism of its initial inaccuracy, Tesla has since provided an explanation of terms and conditions on its website: ‘*Tesla irrevocably pledges that it will not initiate a lawsuit against any party for infringing a Tesla Patent through activity relating to electric vehicles or related equipment for so long as such party is acting in good faith’* [42]. In this case, the pledge is restricted to technologies relating to electric vehicles and is also condition to a non-assertion clause.

Following this, we provide data regarding the distribution of our sample across the patent pledge types and technology areas (see S1 Table for an individual breakdown). 19 pledges (32%) are classified as *conditional*, *open*, 12 as *conditional*, *priced (20%)*, 11 as *conditional*, *restricted (18%)*, 9 as *priced (15%)*, 5 as *open* (8%), 3 as *restricted* (5%), and 1 as *restricted*, *priced* (2%). Our sample did not show patent pledges that can be classified as *conditional*, *restricted*, *priced*. 47 out of 60 patent pledges (78%) relate to Information and Communication Technology (ICT). 6 pledges (10%) can be allocated to technologies that aim at benefiting the environment; 4 (7%) can be assigned to biotechnology and genetics. 3 patent pledges are made by automobile manufacturers, 2 (3%) relate to electric vehicles (Tesla Motors and Ford) and 1 (2%) to fuel-cell vehicles (Toyota).

### The patent licensing taxonomy

**Fig 3:**
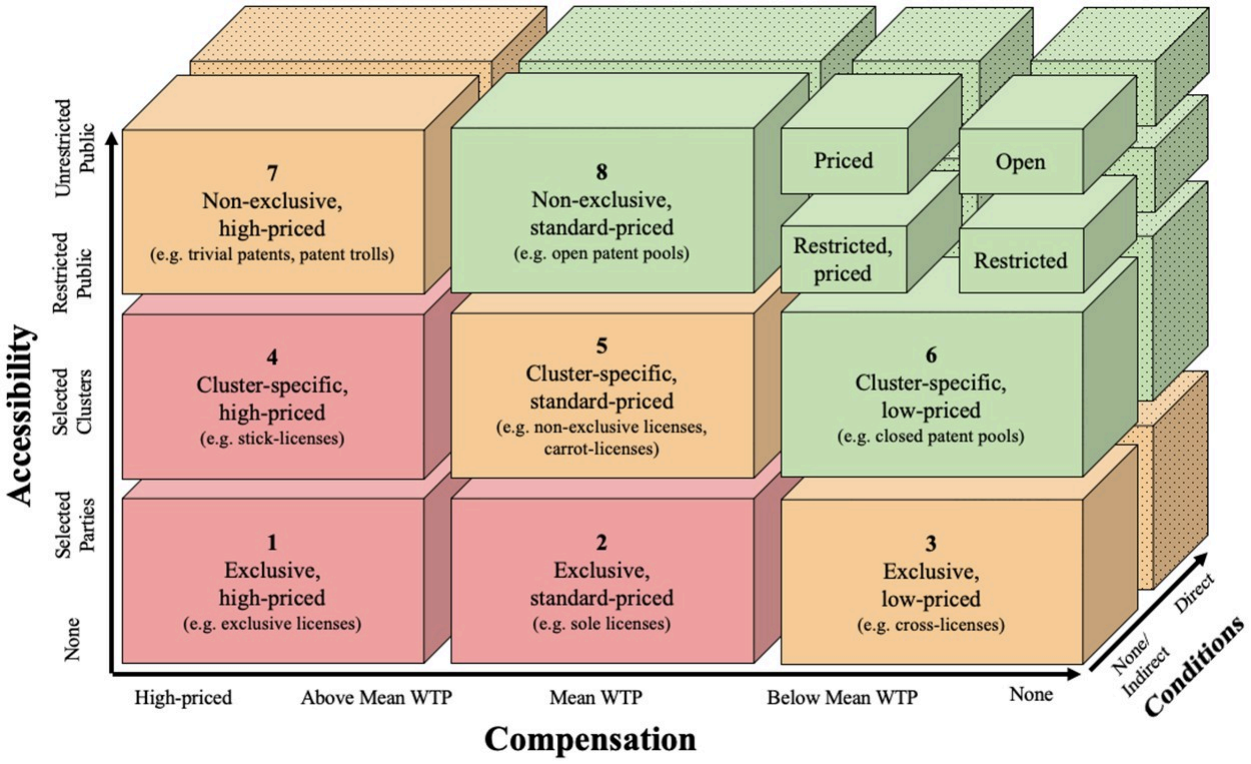
The patent licensing taxonomy.

Academics emphasise that dimensions of reliable taxonomies must be extendable [30]. By examining the patent pledge taxonomy, we find that especially the dimensions *Accessibility* and *Compensation* allow for an extension on one side of the scale. Specifically, there is the possibility that organisations allow very few or no third parties to access their patents. Similarly, they can demand the mean WTP or a higher price as compensation. In contrast, we find no use in extending the dimension *Conditions*, because we have already included the *None* category in the patent pledge taxonomy, and we cannot find more restrictive conditions than the direct restrictions. Therefore, we propose to extend the two dimensions, *Accessibility* and *Compensation*, to categorise patent licensing approaches that are beyond the definition of patent pledges, which then leads to a general patent licensing taxonomy (see Fig 3). In the following section, we describe purposefully, non-randomly selected examples of organisations to substantiate the resulting types [43].

As Fig 3 shows, the dimension *Compensation* has been extended to cover the categories *High-Priced*, *Above Mean WTP*, and *Mean WTP*. It now ranges from high-priced to free of charge. The relationship between FRAND-terms and the WTP, which is the maximal price at which a customer would buy a product or service, has been summarised above [35]. For specific technology areas, certain values for the mean WTP exist. Therefore, the patent licensing taxonomy should be used with regards to specific technologies.

The dimension *Accessibility* has now been extended to cover three additional categories, *Selected Clusters*, *Selected Parties*, and *None*. While the term *Cluster* often refers to the geographical location of firms, we use the term to address a conglomerate of entities defined by the licensor [44]. Therefore, a cluster in the context of the patent licensing taxonomy is a number that falls somewhere between *Selected Parties* and *Restricted Public*. Generally speaking, when an organisation specifies a cluster, it sacrifices some control over the number of potential recipients by addressing a specific group of organisations that can change without the influence of the licensor. This stays in contrast to the category *Selected Parties*, in which the licensor addresses specifically named organisations while retaining full control over the number. However, the number of recipients in a cluster is intended to be smaller than the number referring to the restricted public.

The patent pledge taxonomy with its eight distinct types proposed in the previous section constitutes the upper right cornerstone of the patent licensing taxonomy. The distinction between licensing types regarding their *Accessibility*, *Compensation* and *Conditions* leads to a distinction regarding their *openness* and *exclusiveness*. For instance, *cluster-specific*, *low-priced licensing strategies* in the patent licensing taxonomy are more open with regards to their *Accessibility* and *Compensation* than *exclusive*, *standard-priced licensing strategies*. In contrast, they are more closed regarding their *Accessibility* than patent pledges. Generally, closed approaches in the patent licensing taxonomy are types 1, 2, and 4. Intermediate approaches are types 3, 5, and 7, whilst open approaches are types 6, 8, and all patent pledges. Henceforth, we provide examples for each of the eight remaining types, numbered 1–8, drawing on prior literature.

#### 1 Exclusive, high-priced licensing strategies

*Exclusive*, *high-priced licensing strategies* represent a proprietary approach to an organisation’s patents. Patent owners that employ this approach only share their patents for a high price to carefully selected parties, if at all. Some organisations believe that the exclusion of other parties from their patented invention is the most valuable strategy. We argue that these patent owners would out-license their patents if the price was high enough. However, in many cases they would charge such a high price that interested parties look for other ways to enter the market. This can result in what the academic literature often calls a *monopoly* [45], [46].

Examples for *exclusive*, *high-priced licensing strategies* frequently occur in the pharmaceutical industry. While some patent owners of pharmaceutical drugs license their patents on a high price, most of them refuse to grant a license when there exist no compulsory licensing laws in the respective country [47], [48]. Examples in other industries include Polaroid and Philips. Polaroid excluded its competitor Kodak from the instant camera industry in 1981, and Philips used patents to maintain a monopoly over a specific shaving technology [40], [49]. Generally, exclusive licenses fall under this category.

#### 2 Exclusive, standard-priced licensing strategies

*Exclusive*, *standard-priced licensing strategies* are characterised by a monetary compensation that ranges closely around the mean WTP. Analogous to type 1, the patent owner only allows a few parties to access the respective patents.

Exclusive licenses for different territories, which only allow one party the usage of patents in a specific area, are an example for this type. For instance, the initial polyester patent was exclusively licensed to Du Pont for exploitation in the U.S., while Imperial Chemical Industries (ICI) held the exclusive license for the rest of the world [50]. The territorial restrictions in this example caused the price to go down from high-priced to standard-priced. Licenses in which only one licensee and the licensor are allowed to use the patents are commonly known as *sole licenses*, which are also a form of this type [40].

#### 3 Exclusive, low-priced licensing strategies

*Exclusive*, *low-priced licensing strategies* are accessible to selected parties at a price that is below the mean WTP. Organisations that operate this licensing type carefully select who they allow to use their patents, but the respective patents are also reasonably priced or free of charge.

Hewlett-Packard (HP) is an example of an organisation that uses this licensing strategy. With its wide range of products and a large number of competitors and suppliers, many of HP’s licensing activities in the past did not aim to generate revenue. Rather, its goal was to establish long-term business partnerships with selected parties that built upon low royalty-rates [51]. Generally, open innovation collaborations with common IP-transfers are a further example for *exclusive*, *low-priced licensing strategies* [2]. Lastly, cross-licensing agreements with or without additional royalty-payments also fall into this category. For instance, at one point both Intel and IBM entered a cross-licensing agreement, which automatically licensed a major part of the respective firm’s patents to the other party [52].

#### 4 Cluster-specific, high-priced licensing strategies

*Cluster-specific*, *high-priced licensing strategies* are characterised by licensing-arrangements that offer patents to a cluster of organisations at a price that is above the mean WTP.

In this context, HP provides another example. HP owned patents that were not necessarily of strategic importance to them but that were essential to other organisations of a specific cluster [51]. Here, the firm used out-licensing to generate additional revenue. Yet to keep the price high, the license had not been offered to the restricted or unrestricted public to allow potential licensees to keep a competitive advantage. Hence, HP focused on the monetisation of patents rather than the development of long-term business partnerships which have been described in type number 3. This strategy thus seems to be particularly interesting for patents that are of strategic importance to other organisations. When such organisations involuntarily need to license an invention, the resulting licenses are often referred to as *stick-licenses* [40]. Organisations active in the technology market serve as another example. Qualcomm faced major competition for producing handsets embodying its Code Division Multiple Accessibility (CDMA) technology. To increase revenue, Qualcomm focused on out-licensing to specified clusters rather than producing the technology itself [53].

#### 5 Cluster-specific, standard-priced licensing strategies

*Cluster-specific*, *standard-priced licensing strategies* refer to licensing approaches with patents that are made accessible to a cluster of third parties at a price that is close to the mean WTP.

This licensing type describes an approach in which organisations license their patents to third parties on a common price for this specific cluster [53]. For instance, the Cambridge University spin-off Cambridge Display Technologies (CDT) developed light-emitting plastics and initially tried to manufacture its products on its own [54]. Close to insolvency, CDT changed its business model and out-licensed the technology to entrenched manufacturers on royalty-rates that were common for this industry. CDT needed fast revenues and had no core technology that was essential for other firms. To survive, the firm offered so-called *carrot*-*licenses* in an attempt to incentivise others to buy a license [40].

#### 6 Cluster-specific, low-priced licensing strategies

*Cluster-specific*, *low-priced licensing strategies* differ from patent pledges in the sense that licensors offer the respective patents to a specific cluster only.

Closed patent pools are an example for this licensing type. A closed patent pool consists of at least three parties and allows its members to license patents from other members either for free or at reasonable costs [21], [55], [56]. For instance, the License on Transfer Network (LOT-Network) offers its members both free and reasonable licenses depending on the size of the licensee. LOT was founded by Google, Canon and RedHat and grants its members a license to about 1.2 million IPRs in case those rights are transferred to an NPE (indirect condition). Interested parties must join the LOT-Network and pay an annual fee for the membership. This fee is based on the annual revenue of each firm and ranges from 0 US-Dollars (for firms that generate less than 25 million US-Dollars annually) to a maximum fee of 20.000 US-Dollars (for firms that generate more than 1 billion US-Dollars annually). Furthermore, a limited number of start-ups receive access to royalty-free licenses of three patents of their choice [57]. Importantly, only LOT-members can benefit from these patents.

#### 7 Non-exclusive, high-priced licensing strategies

*Non-exclusive*, *high-priced licensing strategies* refer to the licensing of patents that are offered to the restricted or unrestricted public for a price that exceeds the mean WTP.

The literature suggests that trivial patents which cover multiple constructive technologies can force entire industries into paying high royalty-fees, as the example of Amazon’s 1-click patent from 1999 shows [52], [58], [59]. Examples also include NPEs (often referred to as *Patent Trolls*). NPEs out-license patents to third parties under the threat of litigation if the infringer refuses to buy a license. The threat of an injunction also influences the compensation [58]–[60]. However, as Chesbrough concludes, NPEs can take many forms and seldom use a consistent business model [2]. In sum, *non-exclusive*, *high-priced licensing strategies* can occur as a consequence of trivial patents or due to patent owners with primarily monetary motives that seek to enforce royalty-payments.

#### 8 Non-exclusive, standard-priced licensing strategies

*Non-exclusive*, *standard-priced licensing strategies* are characterised by patents that are available to the restricted or unrestricted public at a price that is close to the mean WTP.

Open patent pools, in contrast to closed patent pools described in type number 6, constitute an example for this licensing approach. Open patent pools provide every interested third party with patent-packages at standard royalty-rates [21], [54], [56]. This is different from closed patent pools in the sense that an open patent pool does not require third parties to become a member. For instance, the patented technologies relating to the Moving Pictures Expert Group 2 (MPEG 2), the 3G Mobile Communication Standards and the Digital Versatile Disk (DVD) were being made available to the unrestricted public at standard royalty-rates through open patent pools [54], [56], [61]. This type includes IPRs that constitute a standard (standard-essential patents or SEPs) and must be made available to the unrestricted public on FRAND-terms. An important characteristic is that they must be offered to the restricted or unrestricted public. Compared to type number 6, this openness often comes at a higher price.

## Discussion

This section will discuss how changes in licensing strategies can be displayed in the patent licensing taxonomy and how the taxonomy can help organisations during strategic decision processes, such as deciding between competing technologies. Furthermore, we will address the limitations of this study and give implications for future research.

### Transitions through the patent licensing taxonomy over time

**Fig 4:**
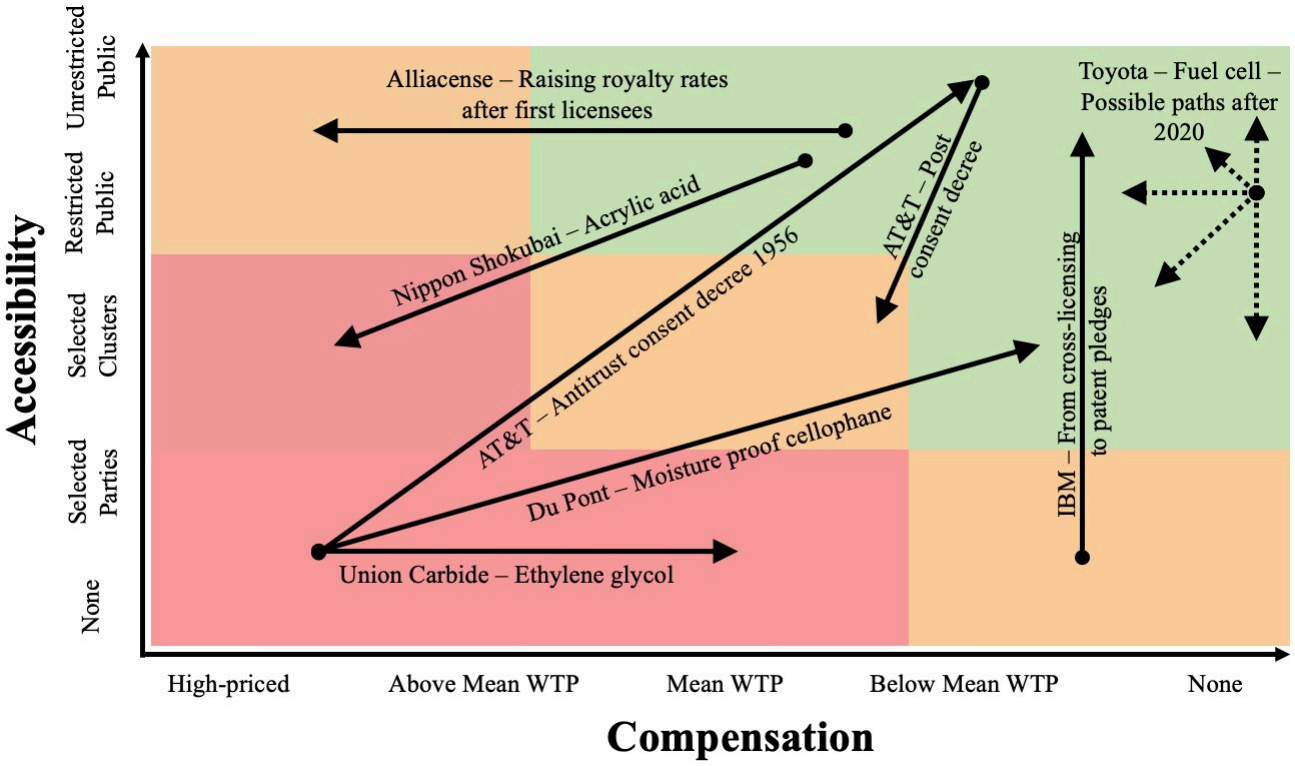
Exemplary paths through the patent licensing taxonomy.

The examples provided in the previous section may suggest that organisations adopt only one licensing type. However, examples show that organisations adapt their licensing strategies over time, and thus transition from one type to another. Furthermore, it is obvious that organisations diversify their licensing strategies for different technologies. In one example, BP Chemicals licensed patents relating to acetic acid only very selectively, whereas it tried to license its patents in the area of polyethylene aggressively [53]. Subsequently, we will describe some case examples for specific changes in openness of licensing and illustrate them in Fig 4.

#### Transitions towards openness

Transitions from exclusiveness towards openness in patent licensing constitute one possibility to adapt licensing approaches over time. For instance, Du Pont held a monopoly for moisture-proof cellophane in the 1930s and allowed only one other company to use the technology under a high-priced license. During that time, Du Pont’s licensing approach was an *exclusive*, *high-priced licensing strategy*. After its competitor Dow Chemicals introduced a new packaging material, Du Pont offered licenses to parties that had previously, without success, requested a license for cellophane. The new licenses had been offered on very reasonable terms in order to enable a quick start and deter Dow Chemical’s entry into this specific market [50]. These new licensing-offers marked a transition of Du Pont’s licensing strategy from *exclusive*, *high-priced* to *cluster-specific*, *low-priced licensing strategies*. Another example is Union Carbide. A major producer of ethylene glycol, the firm initially kept its own production process for glycol secret—these were *exclusive*, *high-priced licensing strategies*. Faced by increasing competition, Union Carbide started to out-license the process selectively and shifted their licensing strategy to *exclusive*, *standard-priced licensing strategies* [62].

Organisations do not necessarily adapt licensing strategies voluntarily. In the first 30 years after its foundation in 1885, AT&T used its patents in an exclusive manner to establish itself in the market. Forced by an antitrust consent decree in 1956, the firm was legally required to license its patents on reasonable royalty-rates below market value [51]. After the consent decree came to an end, AT&T returned to a more closed licensing approach, albeit not as closed as in its formative years.

#### Transitions towards exclusiveness

Organisations can also start with an open approach and adopt a more exclusive licensing approach over time. A reason for this might be the attempt to initially accelerate diffusion and adoption-rates of a patented technology. Once the technology is established, the licensor can return to a proprietary strategy and recover competitive advantages. For example, Toyota’s patent pledge for the royalty-free use of thousands of patents relating to fuel cell vehicles is only valid until 2020. After that, it is possible that the car manufacturer could demand royalty-fees for the use of its patents, thus causing a shift to a more exclusive approach. Over 30 years ago, a similar approach has been described in the context of pricing strategies [63]. *Penetration pricing* is a pricing strategy used in the early stages of a new technology to incentivise as many adopters as possible. Once consumers rely on this adopted technology, the organisation can regain profits by gradually demanding higher prices at a later stage. This might also explain why firms that apply patent pledges do not simply abandon their patents. If they gave up the protection, they could neither demand licensing-fees nor seek injunction at a later stage. Another example of a firm that shifted from open to more exclusive licensing strategies is Nippon Shokubai with its process for producing acrylic acid in the 1970s. As the company changed its business model from licensing patents to the production of acrylic acid, it tightened its attitude by licensing only to markets that it could not supply itself [62].

#### Transitions along single dimensions

Organisations often adapt royalty-rates for their patents to changing competitive conditions and the altered value of its underlying technology [51]. They charge different rates for different licensees or, if permitted in the license agreement, they can change royalty-rates for the same licensee [51]. In one case, the US-firm Alliacense forces third parties into license agreements under the threat of litigations and thus qualifies as a NPE. As some scholars have pointed out, one particularity of this firm is that it offers early licensees’ lower royalty-rates to incentivise rapid license agreements [58]. If an influential company first agrees to the reduced license, others are urged to follow at a higher price [58]. This licensing strategy constitutes the transition from *non-exclusive*, *standard-priced* to *non-exclusive*, *high-priced licensing strategies*. Similarly, patent owners can change the number of potential recipients and can introduce or eliminate conditions for the patent-usage.

### Further discussions and future research

Our findings show that the majority of patent pledges relate to ICT, which is in line with earlier findings [14], [20]. This strengthens the connection between patent pledges, open innovation and open source software [64]. However, since the line between IP-protection and open source software is vague and also country-specific, future research should address this issue by specifically incorporating open source software into the patent licensing taxonomy.

Regarding the distribution of patent pledges, it is noteworthy that *conditional*, *restricted*, *priced patent pledges* did not occur in our sample. This could be a consequence of an organisation’s intention that, when it decides to out-license patents to the restricted public in return for monetary compensation, it does not want to exclude potential licensees by imposing further conditions. While we are confident that both our taxonomies depict practical strategies that are actually used, our results are not definitive. Specifically, we aim to collect further patent pledge data to fill the gap of *conditional*, *restricted*, *priced patent pledges*. Future research should also look towards a more detailed investigation of individual patent pledge types, including the motives behind them and how these relate to specific business models.

Our second taxonomy, the patent licensing taxonomy, can be used as a strategic tool to support certain decisions within an organisation. For instance, when an organisation faces the decision between two competing technologies, it can compare respective licenses for each technology in the patent licensing taxonomy. This provides a profile of the technologies from the perspective of existing licenses, while the strategic importance of individual licenses can be visualised using different shape-sizes. As a result, the organisation gains an overview of the availability and distribution of patents across different owners, which ultimately influences the adoption decision.

Furthermore, the patent licensing taxonomy can uncover connections between firms that apply different business models. For example, whereas Alliacense and Nippon Shokubai are organisations with very different approaches, they both transitioned to a more exclusive licensing approach. The patent licensing taxonomy is a tool that allows for the illustration of multiple licensing strategies within the direct business environment of an organisation. We are aware that the categories of our taxonomies are debatable. To make this illustration more precise and transparent, prospective research should focus on ways to objectively quantify the categories. However, we emphasise that the taxonomies are especially useful when used to compare units of interest. It is easier to position organisations when looking at openness relative to each other, rather than in absolute terms.

## Conclusion

Patent pledges are a phenomenon that has recently gained attention in both the day-to-day business of organisations and in the academic literature. A review of the existing research has shown that the term patent pledges still lacks a clear definition. We believe this is because the consideration of different types of patent pledges has been, to a large extent, overlooked. It is not just patent pledges but most patent licensing approaches that suffer from a lack of coherent definitions, which prohibits a clear distinction between licensing strategies and the comparison of academic research.

In this paper, we have proposed an inductively derived definition of patent pledges, and therefore contribute to the ontology of IP licensing. Furthermore, we have developed two taxonomies, one for patent pledges, and one for patent licensing in general. Both allow for a better distinction of existing licensing strategies in the current business environment and provide a coherent terminology. The patent pledge taxonomy classifies eight distinct types and enables a differentiation between licensing approaches that, until now, have been roughly termed *IP Pledges*. By extending two dimensions of this first taxonomy, we have created the patent licensing taxonomy. In addition to the facilitated distinction between common licensing approaches, the patent licensing taxonomy enables organisations to illustrate and compare the licensing strategies within their business environment, including those of their direct competitors. Both taxonomies can help managers to make informed decisions in the increasingly complex world of patent licensing.

## Supporting information

S1 Table Patent pledge dataset—Types and technology areas. (DOCX)
