# Supplementary material for: Patent pledges, open IP, or patent pools? Developing taxonomies in the thicket of terminologies
Source: PLoS One. 2019 Aug 20;14(8):e0221411. doi: 10.1371/journal.pone.0221411 (PMC6701788; doi:10.1371/journal.pone.0221411)
Supplement: S1 Table — (DOCX) [file pone.0221411.s001.docx]

| **Organisation(s)** | **Country** (Headquarter of organisation) | **Date of press release** | **Number of patents** | **Technology area** | **Patent pledge type** |
| --- | --- | --- | --- | --- | --- |
| Blackboard | United States | 13.03.2010 | 10 | Internet based support systems | Conditional, Open |
| Blockstream | Canada | 19.07.2016 | N/A | Blockchain / Bitcoin | Conditional, Open |
| Eco-Patent Commons | N/A | N/A | 284 | ‘Green’ technology | Conditional, Open |
| Google open patent non-assertion pledge (OPN) | United States | Status: 02.2019 | 202 | Information technology | Conditional, Open |
| GreenXchange (research non-exempt option) | N/A | N/A | N/A | ‘Green’ technology | Conditional, Open |
| GreenXchange (standard plus option / conditions only) | N/A | N/A | N/A | ‘Green’ technology | Conditional, Open |
| iBiquity (free license) | United States | 13.04.2005 | N/A | Standards for transmission equipment that implements NRSC-5 | Conditional, Open |
| IBM | United States | 11.01.2005 | 500+ | Information technology | Conditional, Open |
| IBM | United States | 13.07.2007 | N/A | Specifically named specifications | Conditional, Open |
| IBM | United States | 24.10.2005 | N/A | Healthcare / Education | Conditional, Open |
| Microsoft | United States | 11.2005 | N/A | Microsoft Office 2003 XML reference schemas | Conditional, Open |
| Microsoft | United States | 12.09.2006 | N/A | Various specifications | Conditional, Open |
| Nokia | Finland | 25.05.2005 | N/A | Linux technology | Conditional, Open |
| Open Invention Network (OIN) | United States | Status: 02.2019 | 750+ | Linux technology | Conditional, Open |
| Open Web Foundation (OWF) 1.0 | United States | Status: 11.2018 | N/A | Emerging web technologies | Conditional, Open |
| RedHat | United States | 29.05.2002 | N/A | Software | Conditional, Open |
| Southern California Edison | United States | 2008 | 1 | Method of communicating between a utility and its customer locations | Conditional, Open |
| Sun Microsystems | United States | 30.09.2005 | N/A | Open document standards | Conditional, Open |
| Tesla Motors | United States | 06.2014  Status: 02.2019 | 361 | Electric vehicles and related technology | Conditional, Open |
| Apple | United States | 11.11.2011 | N/A | Cellular technology | Conditional, Priced |
| Google | United States | 08.02.2012 | N/A | All existing licenses after the acquisition of Motorola Mobility Holdings, Inc. | Conditional, Priced |
| GreenXchange (standard plus option/payment and conditions) | N/A | N/A | N/A | ‘Green’ technology | Conditional, Priced |
| Intel | United States | 17.02.2018 | N/A | ‘Industry Standards’ | Conditional, Priced |
| Microsoft | United States | 07.2006 | N/A | Operating system inventions | Conditional, Priced |
| Microsoft | United States | 08.02.2012 | N/A | Standard technologies | Conditional, Priced |
| Microsoft (Interoperability commitment general provisions) | United States | 16.12.2009 | N/A | Public standards in Microsoft’s relevant software products | Conditional, Priced |
| Nokia | Finland | 08.05.2002 | N/A | W-CDMA technology | Conditional, Priced |
| Nokia | Finland | 2010 | N/A | Standards around long-term evolution and service architecture evolution | Conditional, Priced |
| Qualcomm | United States | 12.2008 | N/A | Standards for CDMA-based telecommunication | Conditional, Priced |
| Samsung | South Korea | 27.09.2013 | N/A | UMTS standards | Conditional, Priced |
| Vodafone | United Kingdom | 2017 | N/A | Mobile network technologies | Conditional, Priced |
| iBiquity (FRAND-license) | United States | 13.04.2005 | N/A | Transmission equipment that implements NRSC-5 (no standard) | Restricted, Priced |
| Computer Associates, International | United States | 09.2005 | 14 | Information Technology | Conditional, Restricted |
| Consumer and Merchant Awareness Foundation (CMAF) | N/A | 2013 | 1 | Fraud protection method | Conditional, Restricted |
| Max-Planck-Gesellschaft zur Förderung der Wissenschaften e.V. | Germany | 2006 | 2 | RNA interference mediating small RNA molecules | Conditional, Restricted |
| Microsoft (Interoperability commitment Subject D.) | United States | 16.12.2009 | N/A | Interoperability information | Conditional, Restricted |
| Microsoft | United States | 21.02.2008 | N/A | Technology relating to open source compatibility | Conditional, Restricted |
| Microsoft (Open Specification / Community Promise) | United States | 30.09.2013 | N/A | Specifications for protocols that are used by Windows server operating systems to interoperate with Windows client operating systems | Conditional, Restricted |
| Microsoft (Commitment to Academia) | United States | 03.12.2003 | N/A | Technology relating to web standards | Conditional, Restricted |
| MIT, Max-Planck-Gesellschaft zur Förderung der Wissenschaften e. V., The Whitehead Institute for Biomedical Research, University of Massachusettes, | N/A | 2006 | 11 | RNA sequence-specific mediators of RNA interference | Conditional, Restricted |
| Monsanto | United States | 2014 | N/A | Patented seed and traits | Conditional, Restricted |
| Myriad Genetics | United States | 2014 | N/A | Healthcare | Conditional, Restricted |
| Toyota | Japan | 2015 | 5680 | Fuel cell stacks, high-pressure hydrogen tanks, fuel cell system software control, hydrogen production and supply | Conditional, Restricted |
| Organisations contributing to QR-Code technology | N/A | N/A | 9 | QR-Code technology | Open |
| Gatespace Telematics, IBM, Nokia, ProSyst Software, Samsung | N/A | 26.07.2006 | N/A | OSGi Service Platform Release 4 | Open |
| GreenXchange (standard option) | N/A | N/A | N/A | ‘Green’ technology | Open |
| IBM | United States | 26.09.2006 | 100+ | Business-methods | Open |
| OpenPOWER foundation (RF-mode) | N/A | 06.08.2013 | N/A | Instruction Set Architecture | Open |
| Alcatel-Lucent, Ericsson, NEC, NextWave Wireless, Nokia, Nokia Siemens Networks and Sony Ericsson | N/A | 14.04.2008 | N/A | 3GPP Long Term Evolution and Service Architecture Evolution (LTE/SAE) | Priced |
| OpenPOWER foundation (RAND-mode) | N/A | 06.08.2013 | N/A | Instruction Set Architecture | Priced |
| Ericsson | Sweden | 12.01.2012 | N/A | Mainly wireless technology | Priced |
| Ericsson | Sweden | 27.11.2012 | N/A | Information technology | Priced |
| Ford | United States | 28.05.2015 | 1650 | Electric vehicles and related technologies | Priced |
| GreenXchange (standard plus option / payment only) | N/A | N/A | N/A | ‘Green’ technology | Priced |
| Microsoft | United States | 03.12.2003 | N/A | Clear Type Technology and FAT File system | Priced |
| Microsoft (Open Source Compatibility) | United States | 21.02.2008 | N/A | Technologies relating to Microsoft’s ‘Open Protocols’ | Priced |
| NTT DoCoMo, Ericsson, Nokia, Siemens, Fujitsu, Matsushita Communication Industrial (Panasonic), Mitsubishi Electric, NEC and Sony Corporation | N/A | 06.11.2002 | N/A | W-CDMA technology | Priced |
| Microsoft | United States | 03.12.2003 | N/A | Microsoft Office 2003 XML reference schemas | Restricted |
| Sun Microsystems | United States | 31.01.2005 | 1600 | Technology relating to Sun OpenSolaris | Restricted |
| www.thepatentpledge.org | N/A | Status: 11.2018 | N/A | Software | Restricted |
